# Effects of intensified endurance training on nocturnal skin temperature and sleep variables in male long‐distance runners: A pilot study

**DOI:** 10.14814/phy2.70986

**Published:** 2026-06-30

**Authors:** Saya Okamoto, Kazushige Goto

**Affiliations:** ^1^ Graduate School of Sport and Health Science Ritsumeikan University Kusatsu Shiga Japan

**Keywords:** endurance athletes, endurance training, skin temperature fluctuation, sleep parameters

## Abstract

This study examined the effects of intensified endurance training on nocturnal skin temperature and sleep variables in male long‐distance runners. Fourteen runners completed two different 8‐day training programs: one with intensified training (INT) and one with normal training (NOR). At night, proximal (chest) skin temperature was measured every 5 min. Sleep variables, including efficiency, total time, latency, and wake time after sleep onset (WASO), were assessed for 8 consecutive days. Running distance over 8 days was significantly higher in INT (209.2 ± 17.4 km) than in NOR (118.5 ± 31.2 km, *p* = 0.01). Maximum skin temperature was significantly higher (*p* = 0.045) throughout the night in INT, especially during the first 180 min after bedtime (*p* = 0.03). Delta (Δ) skin temperature (maximum – minimum) during sleep was higher in INT than in NOR (*p* = 0.004). Sleep latency was significantly longer in INT (8.4 ± 4.0 min) than in NOR (2.6 ± 1.9 min, *p* = 0.003). Eight consecutive days of intensified endurance training elevated nocturnal proximal skin temperature with increased sleep latency. These findings suggest that increased training volume may alter nocturnal thermoregulatory responses and delay sleep initiation without substantially impairing overall sleep quantity.

## 1 INTRODUCTION

Endurance athletes experience physiological and psychological stress associated with consecutive days of prolonged exercise, multiple training sessions in a day, and competitions, which can lead to accumulated fatigue with decreased exercise performance (Meeusen et al., 2013). Therefore, it is crucial to ensure adequate recovery to sustain athletic performance and prevent overtraining. However, during intense endurance training (e.g., training camps), athletes often experience disturbed sleep, which may further impair recovery capacity. Thus, it is important to monitor physiological condition continuously, including during rest, to detect early signs of fatigue accumulation and overtraining in endurance athletes (Hickie et al., 2013; Knufinke et al., 2018; Walsh et al., 2021).

Sleep quality is closely related to physiological regulation during the sleep–wake cycle, including thermoregulation and hormonal secretions. Disruption of this rhythm is associated with sleep disturbance and impaired recovery (Czeisler et al., 1999; Kräuchi & Deboer, 2010). Recent advances in monitoring technologies have made it possible to continuously measure core temperature, skin temperature, heart rate, and other physiological parameters. Core and skin temperatures show 24‐h fluctuations, and these changes are closely associated with sleep onset and sleep quality (Refinetti & Menaker, 1992). For instance, an increased distal‐to‐proximal skin temperature (DPG) gradient facilitates peripheral heat loss, thereby promoting sleep onset (Kräuchi & Wirz‐Justice, 2001; Sarabia et al., 2008).

Aloulou et al. (2020) found that high‐intensity, intermittent trail‐running starting at 9 pm and lasting for 48 min increased heart rate and core temperature during the first part of the night, reducing the proportion of rapid eye movement (REM) sleep in trained athletes. However, because core body temperature and skin temperature are not interchangeable, these findings do not clarify nocturnal skin temperature responses, which were not directly assessed in that study. In our previous study, training days were associated with differences in the time course of nocturnal proximal skin temperature during the initial 3 h after bedtime, as well as in total sleep time compared with rest days in long‐distance runners (Okamoto et al., 2026). In other studies, consecutive days of endurance training increased waking time and reduced both total sleep time and subjective sleep quality; however, those studies did not evaluate nocturnal body temperature (Pitchford et al., 2017; Skein et al., 2018). Thus, the impact of consecutive days of endurance training on nocturnal body temperature and sleep parameters remain unknown, particularly under real‐world training conditions.

Therefore, we examined the effects of intensified endurance training on nocturnal body temperature and sleep quality in male long‐distance runners. We compared nocturnal skin temperature and sleep quality between intensified training and normal training. We hypothesized that increased training volume would elevate nocturnal skin temperature and decrease total sleep time.

## 2 METHODS

### 2.1 Participants

Fourteen male long‐distance runners participated in the study. Their (mean ± standard deviation [SD]) age, height, body weight, and body mass index were 19.8 ± 1.0 years, 174.3 ± 4.0 cm, 57.3 ± 4.1 kg, and 18.9 ± 0.9 kg/m^2^, respectively. Participants were recruited from the long‐distance running team of a university track and field club, and all eligible male long‐distance runners who had no injuries or illnesses and agreed to participate were enrolled in the study. All participants provided written informed consent after being fully informed about the aims, procedures, and potential risks of the study. The study was approved by the Ethics Committee for Human Experiments of Ritsumeikan University (BKC‐LSMH‐2024‐008), in accordance with the Declaration of Helsinki.

### 2.2 Experimental design

Skin temperature and sleep variables were monitored continuously during two distinct phases: an 8‐day period in July during the competition season (NOR), and an 8‐day period in September during an intensified training camp (INT). Living arrangements were standardized across conditions, with all participants housed together during both training periods. Daily schedules, including meal timing, bathing, and sleep were not standardized. However, participants were instructed to maintain each activity logs. During NOR, a total of 11 training sessions were performed, including nine jogging sessions ranging from 5 to 20 km and 2 interval training sessions (e.g., 10 × 1000 m). During INT, a total of 21 training sessions were performed, including 14 jogging sessions ranging from 10 to 25 km, 4 interval training sessions, and 3 strength training sessions. During NOR, identical meals were provided to all participants for breakfast and dinner, whereas lunch was individually chosen. During INT, the same meals were served to all participants across breakfast, lunch, and dinner.

### 2.3 Monitoring of skin temperature

Skin temperature was measured continuously every 5 min using a button‐type thermometer (Hal‐Share, SUN･WISE, Osaka, Japan), affixed to the chest area with medical adhesive tape. This sensor has been used in previous studies to monitor 24‐h skin temperature (Shi & Natsume, 2024) and to determine nocturnal skin temperature in menopausal women (Tomiishi et al., 2026). Participants wore the device continuously throughout each period and replaced the tape as needed to prevent rash. The thermometers were attached the day before measurements began, and all data were later extracted and classified according to the daily activity records. From the obtained skin temperature data, the mean, maximum, minimum, and Δ (maximum– minimum) values were calculated for the entire sleep period and the first 180 min after bedtime.

### 2.4 Assessment of sleep variables

Sleep variables (efficiency, total time, latency, and wake time after sleep onset [WASO]) were evaluated using an actigraphy device (wGT3X‐BT, ActiGraph, Pensacola, FL), placed on the non‐dominant wrist. Pitchford et al. (2017) reported that sleep variables can be assessed using actigraphy in participants' home and camp environment. In the present study, the device was worn from 60 min before sleep until waking. Data were stored in 60‐s epochs and analyzed using ActiLife software (version 6.13.5; ActiGraph). Sleep variables were derived using the Cole–Kripke algorithm, with sleep periods defined based on self‐reported sleep logs.

### 2.5 Statistical analyses

Data from 8 of the 14 participants with complete datasets were included in the statistical analyses. Six participants were excluded due to incomplete data collection, including missing skin temperature data (e.g., due to detachment of the skin temperature sensor or failure to wear the actigraph). Results are presented as mean ± SD. To examine changes in skin temperature over time, a two‐way repeated‐measures ANOVA was conducted with condition (INT, NOR) and time (36 points) as factors, testing both main effects and their interaction. For other variables, paired *t*‐tests were used to compare mean values between conditions. Effect sizes were calculated using Cohen's *d* for paired *t*‐tests and partial eta squared (*η* ^2^) for two‐way repeated‐measures ANOVA. Cohen's *d* values were interpreted as trivial (<0.20), small (0.20–0.49), moderate (0.50–0.79), and large (≥0.80). For partial eta squared (*η* ^2^), values of 0.01, 0.06, and 0.14 were considered small, moderate, and large effects, respectively (Cohen, 1988). A *p* value <0.05 was considered statistically significant for all analyses. Statistical analyses were performed using SPSS software (version 28.0; IBM Corp., Armonk, NY, USA).

## 3 RESULTS

### 3.1 Body composition

Table 1 summarizes the data on body composition. No variables significantly differed between INT and NOR.

**TABLE 1:** Comparisons of body composition over experiment period.

	NOR	INT	*p* Value (effect size)
Height (cm)	173.6 ± 3.6	—	—
Body weight (kg)	56.4 ± 3.5	56.1 ± 3.7	0.30 (*d* = 0.08)
Body fat (kg)	6.0 ± 1.1	5.3 ± 1.0	0.11 (*d* = 0.67)
% Body fat (%)	10.6 ± 2.0	9.4 ± 1.8	0.09 (*d* = 0.63)

*Note*: Values are mean ± SD.

### 3.2 Running distance

Total running distances were significantly longer during INT (209.2 ± 17.4 km) than during NOR (118.5 ± 31.2 km, *p* = 0.01).

### 3.3 Skin temperature during sleep

Table 2 summarizes skin temperatures during sleep during INT and NOR. Maximum temperature was significantly higher throughout the night (*p* = 0.045) and during the first 180 min after bedtime (*p* = 0.03) in INT compared to NOR. Δ skin temperature was also elevated during INT across the entire night (*p* = 0.004) but not within the first 180 min. No other variables differed significantly between conditions.

**TABLE 2:** Skin temperature during sleep in NOR and INT.

	NOR	INT	*p* Value (effect size)
Mean (°C)	33.94 ± 0.43	34.06 ± 0.66	0.54 (*d* = 0.22)
180 min after bedtime	34.01 ± 0.45	34.25 ± 0.79	0.31 (*d* = 0.37)
Maximum (°C)	35.41 ± 0.59	35.78 ± 0.50*	0.05 (*d* = 0.68)
180 min after bedtime	34.53 ± 0.53	34.92 ± 0.57*	0.03 (*d* = 0.71)
Minimum (°C)	32.69 ± 0.54	32.15 ± 0.63	0.06 (*d* = 0.92)
180 min after bedtime	33.48 ± 0.53	33.53 ± 0.84	0.90 (*d* = 0.07)
ΔTsk (°C)	2.72 ± 0.57	3.63 ± 0.34*	0.004 (*d* = 1.94)
180 min after bedtime	1.05 ± 0.42	1.39 ± 0.35	0.22 (*d* = 0.88)

*Note*: Values are mean ± SD. Skin temperature during sleep was extracted from the overall data based on the sleep time recorded by the participants. ΔTsk was calculated by subtracting minimum of Tsk from the maximum of Tsk.

* *p* < 0.05 versus NOR.

**FIGURE 1:**
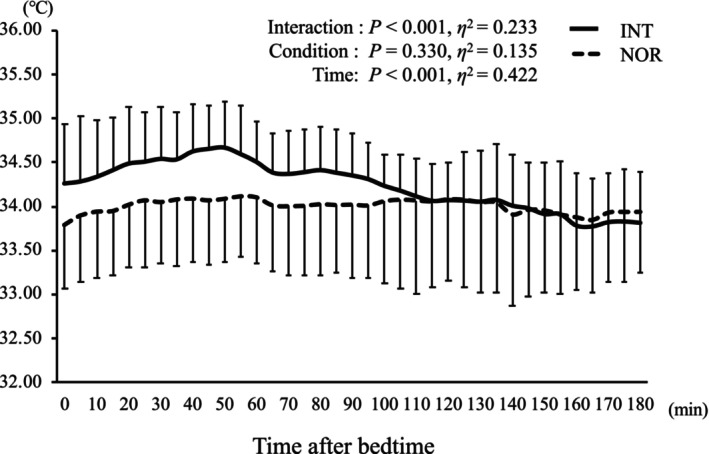
Skin temperature fluctuation during initial 180 min after bedtime. Values are mean ± SD.

Figure 1 illustrates the fluctuations in skin temperature during the first 180 min after bedtime, a period that was emphasized because nighttime exercise alters early sleep thermoregulation (Aloulou et al., 2020). A significant condition × time interaction (*p* < 0.001, *η* ^2^ = 0.233) and a main effect of time (*p* < 0.001, *η* ^2^ = 0.422) were observed.

### 3.4 Sleep variables

Table 3 presents the sleep variables during each training period. Only sleep latency differed significantly between the two training periods, being longer during INT (*p =* 0.003).

**TABLE 3:** Sleep parameters in NOR and INT.

	NOR	INT	*p* Value (effect size)
Sleep efficiency (%)	85.2 ± 7.2	83.7 ± 5.1	0.19 (*d* = 0.24)
Total sleep time (min)	360.0 ± 21.8	381.0 ± 25.5	0.07 (*d* = 0.89)
Sleep latency (min)	2.6 ± 1.9	8.4 ± 4.0*	0.003 (*d* = 1.85)
WASO (min)	61.2 ± 32.6	65.8 ± 23.1	0.44 (*d* = 0.16)

*Note*: Values are mean ± SD.

Abbreviation: WASO, wake time after sleep onset.

* *p* < 0.05 versus NOR.

## 4 DISCUSSION

We examined the effects of 8 consecutive days of endurance training at different training volumes on nocturnal skin temperature and sleep variables in male long‐distance runners. Skin temperature during the first 180 min after bedtime was significantly higher in INT than in NOR. Moreover, an increased training volume partially affected sleep latency. However, sleep efficiency, total sleep time, and WASO did not differ significantly between conditions. These results suggest greater training volume elevates nocturnal thermoregulatory responses and delays sleep initiation in endurance athletes.

Maximum skin temperature during sleep was significantly elevated during INT, while the minimum temperature remained unchanged, resulting in a greater Δ skin temperature. This supports the conclusion that increased training volume influences nocturnal thermoregulation. In previous studies, high‐intensity intermittent trail running at night increased nocturnal core temperature and reduced REM sleep (Aloulou et al., 2020), and 4 consecutive days of intermittent cycling in the evening attenuated the nocturnal decline in core temperature and decreased REM sleep (Yamanaka et al., 2015). However, those studies determined the effects of a single session of exercise under laboratory‐based controlled conditions. In contrast, we monitored nocturnal skin temperature and sleep quality across 8 consecutive days in real training environments and found that repeated endurance training modified nocturnal temperature regulation.

It should be noted that distal skin temperature and core temperature were not measured; skin temperature was assessed only at a proximal site on the chest. Nevertheless, the elevated skin temperature observed may reflect residual heat accumulation induced by consecutive days of daytime endurance training (INT: 209.2 ± 17.4 km over 8 days). Supporting this interpretation, previous studies have reported that endurance exercise performed close to bedtime increased nocturnal core temperature around sleep onset (Aloulou et al., 2020; Yamanaka et al., 2015; Youngstedt, 2005).

Sleep latency significantly increased during INT. This measure reflects the ease of transitioning into sleep (Kräuchi & Wirz‐Justice, 2001), and the delay observed here can be explained by elevated skin temperature during the initial phase after bedtime. Normally, core temperature decreases while distal skin temperature increases to promote heat loss, facilitating sleep onset (Kräuchi & Wirz‐Justice, 2001; Van Someren, 2006). However, because the distal–proximal skin temperature gradient (DPG) was not measured in the present study, the contribution of thermoregulatory heat loss to sleep onset could not be directly determined. Proximal skin temperature (e.g., chest) typically shows a transient increase at sleep onset followed by a gradual decline, reflecting efficient thermoregulatory adjustments as core temperature continues to decrease (Van Someren, 2006). In the present study, however, INT resulted in a significantly higher proximal chest skin temperature for the first 180 min after bedtime compared to NOR, suggesting that the normal decline was attenuated.

High‐intensity endurance exercise elevates heart rate and core temperature for several hours, potentially interfering with the physiological processes that facilitate sleep onset (Aloulou et al., 2020). Because skin temperature fluctuations during the first 180 min after bedtime differed between the conditions, altered thermoregulatory responses in the early phase of sleep likely contributed to the delayed sleep initiation observed during INT, consistent with previous findings (Kräuchi & Wirz‐Justice, 2001; Van Someren, 2006; Youngstedt, 2005).

Several limitations of this study should be acknowledged. First, the sample size was small, as data from only eight participants were collected during both INT and NOR. Nevertheless, the primary outcomes showed large effect sizes (e.g., *d* = 1.94 for ΔTsk, *d* = 0.68 for maximum skin temperature, *d* = 1.85 for sleep latency, η^2^ = 0.23 [interaction] for change in nocturnal skin temperature), implying that the differences between INT and NOR were reliable despite the limited sample. Second, skin temperature was measured only at a proximal site (chest), and neither core body temperature nor distal skin temperature was assessed. Thus, the present study may not have fully captured thermoregulatory responses relevant to sleep onset. Although the DPG is required to evaluate heat dissipation, it could not be calculated because distal skin temperature was not measured. Third, because the study was conducted under real training conditions, daily routines such as meal timing, bathing, and sleep behaviors were not fully standardized between INT and NOR. In addition, although training volume differed between conditions, subjective fatigue and objective parameters (e.g., running pace and heart rate during each training session) were not assessed. Therefore, the extent to which differences in daily routines and individual training volume influenced nocturnal skin temperature and sleep variables could not be fully determined. Fourth, ambient temperature during sleep was neither standardized nor recorded. Although participants were able to sleep in environments that were individually comfortable, the potential influence of environmental temperature on nocturnal skin temperature cannot be completely excluded. Fifth, the order of conditions was not counterbalanced (NOR preceded INT), raising the possibility of order effects such as seasonal differences or time‐dependent adaptations. However, training volume during the 2 months prior to INT was comparable to NOR, making pre‐adaptation unlikely. Moreover, although mean daytime ambient temperature differed by approximately 10°C between the two periods (INT: 15.3°C; NOR: 24.8°C), nocturnal skin temperature was elevated only during INT, suggesting that training volume rather than seasonal variation was the primary factor. Sixth, all participants were male long‐distance runners, therefore, the findings may not be generalizable to female athletes or to middle‐aged and older populations. Finally, sleep was assessed via actigraphy rather than polysomnography, and therefore detailed sleep architecture could not be evaluated. Future studies should include larger samples, incorporate female athletes, and employ more comprehensive monitoring (e.g., core temperature, heart rate variability, sympathetic nervous system activity, and hormonal responses) to clarify the mechanisms linking increased training volume, thermoregulation, and sleep.

## 5 CONCLUSION

In this pilot field‐based study in male long‐distance runners, 8 consecutive days of intensified endurance training was associated with elevated nocturnal skin temperature and prolonged sleep latency compared to training at normal volume. In contrast, sleep efficiency, total sleep time, and WASO did not differ between conditions. These findings suggest that increased training volume may alter nocturnal thermoregulatory responses and delay sleep onset without substantially impairing overall sleep quantity. Monitoring nocturnal skin temperature may therefore serve as a practical, noninvasive approach for detecting subtle physiological changes in endurance athletes during periods of intensified training.

## AUTHOR CONTRIBUTIONS

**Saya Okamoto:** Conceptualization; data curation; formal analysis; investigation; methodology. **Kazushige Goto:** Conceptualization; project administration; supervision.

## FUNDING INFORMATION

This study was supported in part by JST SPRING, Grant Number JPMJSP2101.

## CONFLICT OF INTEREST STATEMENT

The authors declare no conflict of interest and have no financial relationship to disclose.

## ETHICS STATEMENT

The study protocols were approved by the Ethics Review Committee for Medical and Health Research Involving Human Subjects at Ritsumeikan University, Japan (Approval No. BKC‐LSMH‐2024‐008). All procedures were conducted in accordance with the Declaration of Helsinki.

## Data Availability

The data and analyses that support the findings of this study are not publicly available. The datasets are available from the corresponding author on reasonable request.
